# Association between peri‐operative red blood cell transfusion and cancer recurrence in patients undergoing major cancer surgery: an umbrella review*

**DOI:** 10.1111/anae.16501

**Published:** 2025-01-08

**Authors:** Joshua Etheridge, Panth Shah, Simon J. Stanworth, Ewen Harrison, Michael Gillies, Timothy S. Walsh, Akshay Shah

**Affiliations:** ^1^ Department of Anaesthesia Pain and Critical Care Medicine, Royal Infirmary of Edinburgh Edinburgh UK; ^2^ Usher Institute of Population Health Sciences, The University of Edinburgh Edinburgh UK; ^3^ Department of Medicine University of Saskatchewan Saskatoon Canada; ^4^ NIHR Blood and Transplant Research Unit in Data Driven Transfusion Practice University of Oxford Oxford UK; ^5^ Department of Haematology Oxford University Hospitals NHS Foundation Trust Oxford UK; ^6^ Centre for Medical Informatics University of Edinburgh Edinburgh UK; ^7^ Nuffield Department of Clinical Neurosciences University of Oxford Oxford UK; ^8^ Department of Anaesthesia, Hammersmith Hospital Imperial College Healthcare NHS Trust London UK

**Keywords:** cancer, peri‐operative care, red blood cells, transfusion

## Abstract

**Introduction:**

Peri‐operative allogeneic red blood cell transfusion is hypothesised to increase the risk of cancer recurrence following cancer surgery. However, previous data supporting this association are limited by residual confounding. We conducted an umbrella review (i.e. a systematic review of systematic reviews) to synthesise and evaluate the evidence between red blood cell transfusion and cancer recurrence.

**Methods:**

We searched online databases for systematic reviews of red blood cell transfusion and cancer‐related outcomes. The AMSTAR 2 tool was used for quality assessment. The adequacy of confounding adjustment was judged according to a consensus‐derived framework.

**Results:**

We included five relevant systematic views which included patient populations ranging from 2110 to 184,190. Two reviews reported cancer recurrence, and all reported an association with red blood cell transfusion. Three reviews reported positive associations between red blood cell transfusion and adverse outcomes including all‐cause mortality, recurrence‐free survival and cancer‐related mortality. According to AMSTAR 2, four reviews were rated as ‘critically low quality’ and one as ‘low quality’. There was variation in how systematic reviews assessed the risk of bias from confounding. Compared with our pre‐derived framework, we found a high likelihood of unmeasured confounding.

**Discussion:**

Currently available evidence describes an association between peri‐operative red blood cell transfusion and cancer recurrence, but this is mostly of low to critically low quality, with minimal control for residual confounding. Further research, at low risk of bias, is required to provide definitive evidence and inform practice.

## Introduction

Red blood cell (RBC) transfusion is used to treat anaemia in patients undergoing major surgery. Systematic reviews, meta‐analyses and clinical guidelines generally support restrictive RBC use with transfusion ‘triggers’ of 70–80 g.l^‐1^ [1]. However, there is potential heterogeneity of treatment effect in different populations and/or at different stages of an illness, with limited evidence for many specific populations. Red blood cell transfusion may cause transfusion‐related immunomodulation – a down‐regulation of the recipient's cellular immunity and immune surveillance systems – which could predispose to cancer recurrence and other adverse outcomes associated with RBC transfusion (e.g. infection) [2].

**Figure 1:**
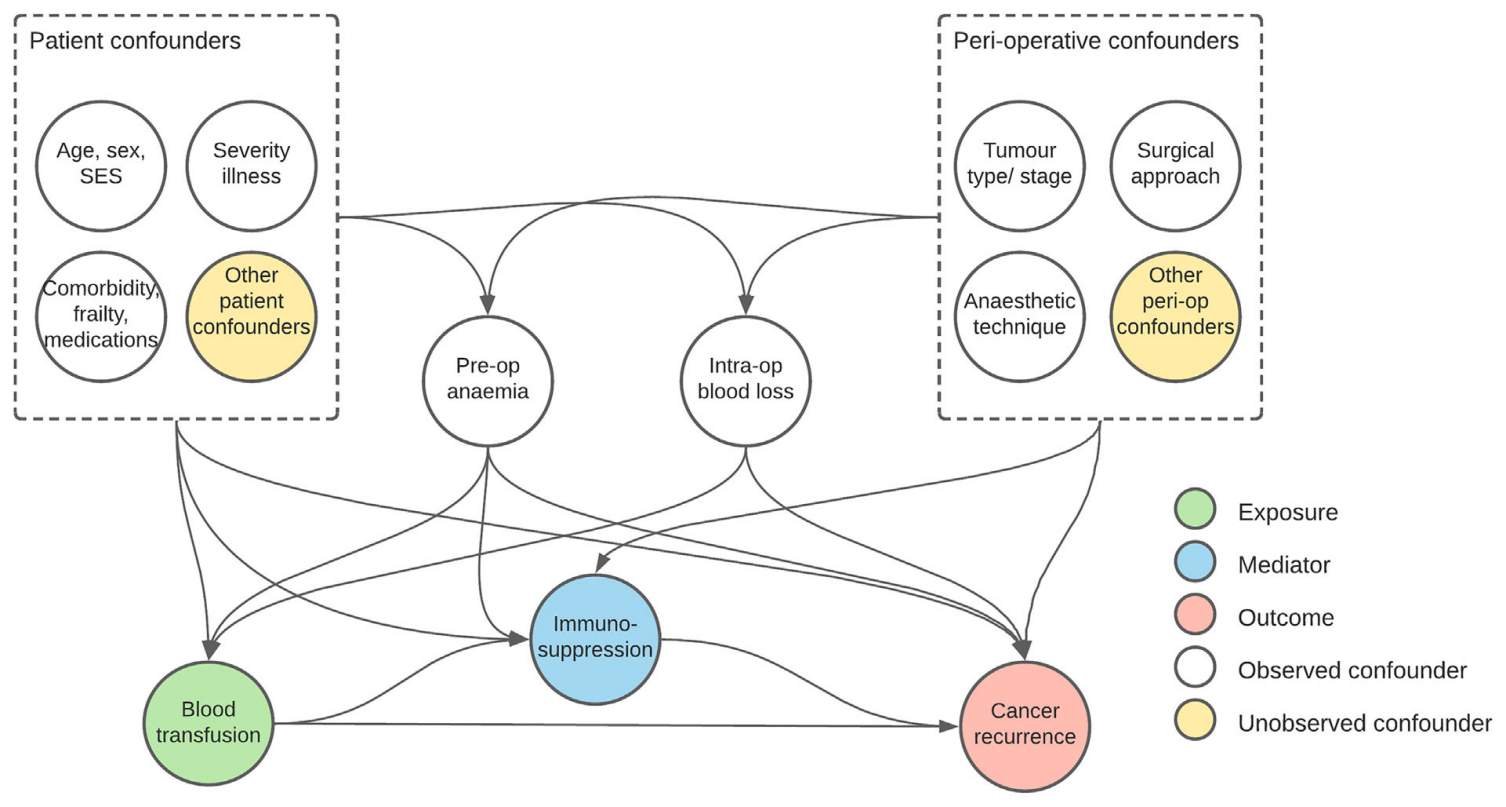
Simplified direct acyclic graphs illustrating the causal relationship between peri‐operative red blood cell transfusion and cancer recurrence. Immunosuppression is hypothesised to be the key mediator of transfusion on cancer recurrence. SES, socioeconomic status; pre‐op, pre‐operative; intra‐op, intra‐operative; peri‐op, peri‐operative.

Early observational studies, mainly in patients undergoing colorectal cancer surgery, reported associations between RBC exposure, cancer recurrence and reduced long‐term survival [3]. Many improvements to RBC production, transfusion practice, general peri‐operative care and cancer treatments have occurred in recent years. These include RBC leucoreduction; blood management programmes; and developments in surgical and anaesthetic practice, which have decreased peri‐operative RBC use [4, 5]. The poor outcomes observed in earlier studies could also be attributed to advanced patient age; comorbidities; pre‐operative anaemia; and difficulty and duration of surgery [6]. Therefore, the observed associations may be as a result of uncontrolled/unmeasured confounding (Fig. 1), and it is plausible that the risk‐to‐benefit balance for RBC transfusion has changed.

Surveys show variation in practice in postoperative RBC transfusion following cancer surgery [7]. This is relevant to enhanced recovery after surgery programmes, as untreated anaemia may contribute to delayed recovery, for example through fatigue or exacerbation of comorbidities such as cardiovascular disease. The prevalence of anaemia is likely to increase because of adherence to restrictive transfusion policies recommended by guidelines [8]. As anaemia is also associated with increased morbidity and mortality [9, 10, 11], there is a need to review the quality of current evidence regarding RBC transfusion for patients undergoing cancer surgery.

## Methods

We followed Cochrane recommendations [12] and reported our findings according to the development of the PRIOR statement [13]. We searched MEDLINE, PubMed, Embase, Cochrane Database of Systematic Reviews and the Transfusion Evidence Library. No restrictions were imposed on publication date. An initial search was conducted in November 2022 and updated again in August 2024. We restricted studies to English language publications. The complete search strategy is reported in online Supporting Information Appendix S1. Literature searches were uploaded to Covidence and two reviewers (JE and PS) screened study titles and abstracts independently for eligibility. The same two reviewers screened full text reports independently for inclusion. Disagreements were resolved through discussion, with arbitration by a third reviewer (TW) if required.

We included systematic reviews which contained data on adults (aged ≥ 16 y) undergoing curative surgery for primary solid organ cancers and requiring any volume of RBC transfusion for the treatment of peri‐operative anaemia. Studies were excluded if the stated population was patients in whom the main cancer management was not surgery alone, and if the intervention under investigation included blood products other than RBCs. Two authors (JE and PS) performed data extraction from selected studies independently onto pre‐piloted forms, with cross‐referencing of the data sets produced to resolve potential errors. Data extracted included author; publication date; number of included studies; study types included; number of patients in the control and intervention groups; and the type of cancer(s) studied.

Our primary outcome of interest was cancer recurrence. Secondary outcomes included recurrence‐free survival; all‐cause mortality; cancer‐related mortality; and peri/postoperative complications as defined by the respective studies, including postoperative infection and surgical re‐intervention.

The quality of each included review was assessed using the AMSTAR 2 tool [14]. During the development of our study protocol, it became clear that most risk‐of‐bias tools, including AMSTAR 2, do not provide in‐depth consideration of the possibility of unmeasured or residual confounding from a clinical perspective. This was thought to be especially relevant to this review given the potential for many potential confounders (Fig. 1). Therefore, in addition to reporting the AMSTAR 2 score, we developed a framework for structured evaluation of potential confounders in the included review. We listed all potential confounding variables they considered important from our clinical perspectives namely: surgery (EH); anaesthesia/critical care (MG, TW and AS); and transfusion medicine (SS). Through joint consensus we developed a framework of potential confounders to the relationship between exposure (blood transfusion) and outcomes (cancer recurrence; survival) following cancer surgery (Table 1). For each included study (when undertaken), we assessed whether any potential confounding variables were identified, whether adjustments were made for the identified confounders and the extent to which adjustment for confounders was undertaken against this consensus framework. We also explored qualitatively whether there was any association between the magnitude and direction of any observed effect and the number of confounding variables adjusted for in the analyses. We performed a narrative analysis of the results for the included reviews relating to our outcomes of interest.

**Table 1:** Consensus framework of potential confounding variables in patients with solid organ cancer, undergoing curative surgery, as identified by expert panel.

Patient factors	Disease factors	Surgical factors	Transfusion factors
Age ASA physical status Comorbidities Frailty BMI Sex Smoking status Socio‐economic status Weight loss	Cancer type Contamination (e.g. perforated cancer) Pre‐operative anaemia Pre‐operative neoadjuvant chemo/radiotherapy Presentation (symptomatic, screening, incidental) Site Tumour node metastasis staging	Anaesthetic technique Approach (e.g. open vs. minimally invasive) Estimated blood loss Intent (e.g. palliative vs. curative) Intra‐/postoperative complications Margin status Operative experience Surgical urgency	Co‐interventions (e.g. tranexamic acid, cell salvage) Document use of patient blood management bundle Leucodepletion Number of red blood cell units transfused Storage age of red blood cells Transfusion thresholds

## Results

**Figure 2:**
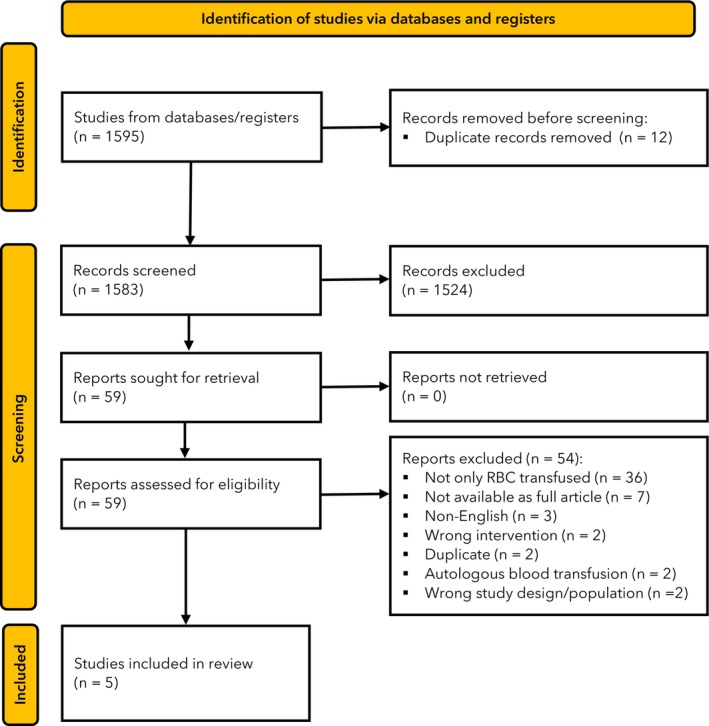
Study flow diagram. RBC, red blood cell.

**Figure 3:**
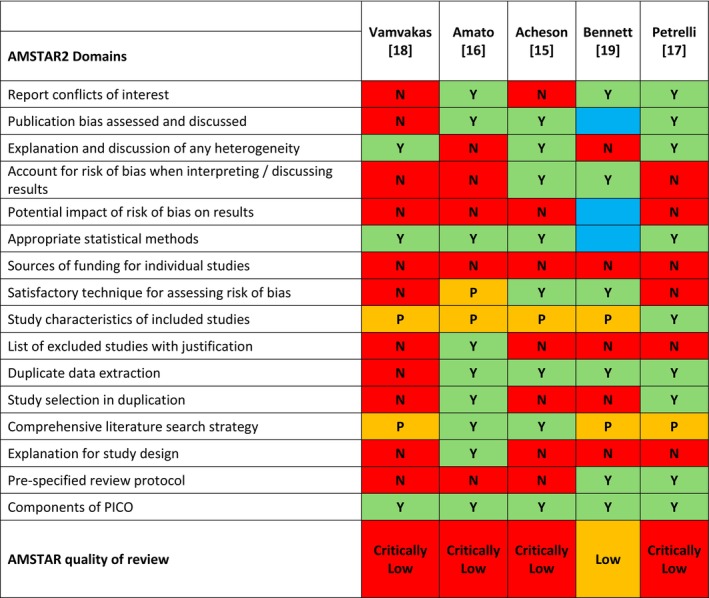
AMSTAR 2 assessment of study quality. Y, yes; N, no; P, partial yes; Blank, not relevant to this study.

The search identified 1595 articles. After screening, we assessed 59 full‐text articles, of which five were suitable for inclusion (Fig. 2). A summary of the five included systematic reviews is shown in Table 2 with detailed results in online Supporting Information Table S1. Of the five studies, four [15, 16, 17, 18] were systematic reviews and meta‐analyses, and one [19] was a systematic review alone. The study period ranged from 1985 [15, 18] to 2019 [17], and included studies analysed between seven [16] and 123 [17] primary data sources. The number of participants ranged from 2110 [15] to 184,190 [17]. Two studies [15, 16] analysed data for colorectal cancer only. Three studies [17, 18, 19] analysed data on different solid organ cancers. Four studies [15, 17, 18, 19] reported data on cancer‐related effects of RBC transfusion alone, while one [16] presented this as a subgroup analysis alongside the effects of administration of other blood products. Only two of the studies summarised follow‐up periods with one [15] reporting a mean (SD) observation time of 62.5 (28.8) months and one [17] reporting a range of median follow‐up times from 10 to 99.5 months. Only one study [15] described whether the RBCs used in the included studies were leucodepleted vs. non‐leucodepleted. None of the studies included a clear definition of ‘peri‐operative period’. According to AMSTAR 2, four reviews were rated as ‘critically low quality’ [15, 16, 17, 18], and one as ‘low quality’ [19] (Fig. 3). A summary of the approach to risk of bias assessment, quality assessment and confounding variable adjustment in each included study is shown in online Supporting Information Table S2.

**Table 2:** Characteristics of systematic reviews included in the umbrella review.

Study	Individual studies included	Publication period of studies	Included study types	Types of cancer included	Number of patients			Key findings
					Total	Received transfusion	No transfusion	
Vamvakas [18]	60	1985–1994	Prospective cohort; retrospective cohort	Colorectal; breast; head and neck; lung; prostate; gastric	19,387	9417	9970	Statistically significant deleterious effect transfusion effect in all cancer sites, except breast
Amato [16]	7	1989–2001	Prospective cohort; retrospective cohort; RCT	Colorectal	2110	1016	1094	Moderate association between RBC transfusion and increased risk of recurrence of colorectal cancer
Acheson [15]	55	1985–2008	Prospective cohort; retrospective cohort	Colorectal	20,795	12,242	8553	RBC transfusion was associated with adverse clinical outcomes, including mortality
Bennet [19]	22	1992–2015	Retrospective cohort	Hepatocellular; colorectal metastases; cholangiocarcinoma	6832	2617	4215	RBC transfusion was associated with worse outcomes including postoperative complications and long‐term cancer recurrence
Petrelli [17]	123	1989–2019	Observational cohort; RCT	Genitourinary; gastrointestinal; thoracic; gynaecological; head and necks sarcoma	184,190	NR	NR	RBC transfusions lowered survival rates and increased risk of cancer recurrence

RCT, randomised controlled trial; NR, not reported; RBC, red blood cell.

Vamvakas explored the association between RBC transfusion and cancer recurrence by tumour site and reported positive associations in colorectal (relative risk (RR) (95%CI) 1.49 (1.23–1.79)); head and neck (RR (95%CI) 3.62 (2.15–6.08)); lung (RR (95%CI) 1.30 (1.02–1.66)); prostate (RR (95%CI) 1.51 (1.13–2.01)); and gastric (RR (95%CI) 2.11 (1.60–3.71)) cancers [18]. There was no evidence of an effect in patients with breast cancer (RR (95%CI) 1.06 (0.90–1.24)). There was statistically significant heterogeneity in the meta‐analysis for the colorectal and gastric cancer groups. The authors did not conduct an evaluation of study quality or risk of bias, and did not consider confounding adjustment in the included studies or overall meta‐analysis.

Amato et al. presented data on the association between cancer recurrence and RBC transfusion as a subgroup analysis of seven studies within a wider systematic review [16]. They reported an association between RBC transfusions and cancer recurrence (odds ratio (OR) (95%CI) 1.65 (1.35–2.01)). They evaluated study quality using a method including 33 questions, with a maximum achievable score of 100 [20] and reported an average score of 66 across all studies. Of the seven studies, three were reported as ‘low risk’ of bias and four as ‘unclear risk’. Most studies were small with an average of 337 participants (range 33–961). The authors noted potential confounding variables from all four of our pre‐defined framework domains, including patient characteristics and comorbidities; pre‐operative anaemia; duration of operation; volume of blood loss; and amount, type, and timing of peri‐operative RBC transfusion. They undertook stratified meta‐analyses when data were available. The authors explored potential effects for several confounding variables on outcomes, for example the timing of RBC transfusion, but it was not clear what confounding adjustment was undertaken relevant to our study question.

Acheson et al. found an association between peri‐operative RBC transfusion and all of their reported outcomes, including all‐cause mortality (OR (95%CI) 1.72 (1.55–1.91)); cancer‐related mortality (OR (95%CI) (1.43–2.05)); postoperative infection (OR (95%CI) 3.27 (2.05–5.20)); and surgical re‐intervention (OR (95%CI) 4.08 (2.18–7.62)) [15]. They reported study quality using the Newcastle‐Ottawa score [20], with a score of 7 as the cut‐off to determine study quality (≤ 7 low‐quality, ≥ 8 high‐quality). Twenty‐eight studies were judged to be of low quality and 27 as high quality. The authors identified confounding variables from three of our four pre‐defined categories, namely patient, disease and transfusion related factors, but did not consider the influence of surgical factors. However, despite identifying potentially relevant confounders, confounder adjustment was not performed due to lack of access to individual patient data.

Bennett et al. conducted a systematic review with narrative synthesis. One of five studies showed increased mortality, 10 of 18 studies showed decreased cancer‐related survival and five of six studies showed increased postoperative complications in patients receiving peri‐operative RBC transfusion [19]. The authors used the Cochrane Risk of Bias Assessment Tool: for Non‐Randomized Studies of Interventions to describe risk of bias among included studies. None were classified as ‘low risk’; 18 were ‘moderate risk’; one was ‘serious risk’; and three were ‘critical risk’ of bias. The ‘critical risk’ of bias papers were not used by the authors in their final analysis. Not all studies adjusted for confounders, and variability regarding confounding variables was considered. Confounding variables in individual studies included surgical technique; patient age; patient sex; comorbidities; volume of blood loss; tumour characteristics (size, grade); vascular invasion; duration of surgery; year of surgery; extent of resection; and other tumour specific variables. There was variation in confounding adjustment across the included studies.

Petrelli et al. published the largest meta‐analysis which included 123 individual studies [17]. Pooled analyses showed an increased hazard ratio in overall survival (hazard ratio (95%CI) 1.50 (1.42–1.57)) and disease‐free survival (hazard ratio (95%CI) 1.36 (1.26–1.46)) in patients who received a peri‐operative RBC transfusion. Using the Newcastle‐Ottawa Score [21] to assess study quality, 21 (17.1%) of studies scored 5 (lowest score); 22 (17.9%) scored 8 or 9; and the remaining 80 (65%) scored 6 or 7. Assessment as low risk of bias in primary studies depended on three elements: adjusting for at least three variables (from age; sex; cancer stage; haemoglobin; and ASA physical status or performance status); adequate follow‐up duration; and data on how the volume/timing of transfusions was assigned. With this assessment, 102 (83%) of included studies were described as having either a ‘low’ or ‘medium’ risk of bias. The ‘trim and fill method’ was also used to assess small studies and the effect of publication bias [22]. In the meta‐analysis, the authors did not stratify their analysis for confounding variables but highlighted which studies adjusted for variables limited to patient age; sex; cancer stage; haemoglobin levels; ASA physical status; and performance status. This included variables from only two of our four pre‐defined categories of confounding variables (patient factors and disease factors). They noted variability within included studies regarding confounding adjustment, but did not address this in detail in the interpretation of their data.

## Discussion

In our umbrella review exploring the relationship between peri‐operative RBC transfusion and cancer‐related outcomes following cancer surgery we found evidence of an association between RBC exposure and adverse cancer‐related outcomes. However, critical appraisal using the AMSTAR 2 tool rated four of the five included systematic reviews as ‘critically low quality’ and one as ‘low quality’. There was substantial unmeasured and residual confounding to the associations described in individual studies and from integration in systematic reviews.

The relationship between RBC transfusion and clinical outcomes is complex in most clinical settings. Interpretation of observational research is especially difficult for multiple reasons. Anaemia, including anaemia severity, is associated independently with adverse outcomes in the peri‐operative period in most surgical populations [23, 24, 25]. It is therefore difficult to discriminate risk attributable to anaemia from risk from RBC transfusions as a treatment for this condition. Patients with pre‐operative anaemia are more likely to have pre‐existing ill health and comorbidities, which needs adequate consideration and adjustment. Patients who develop peri‐operative anaemia are more likely to have experienced greater surgical blood loss, which could be because they had more advanced disease or required more extensive or complex surgery. All these factors can confound the association between RBC transfusions and outcomes, including for cancer surgery. Furthermore, anaemia is more prevalent in patients who experience complications that can affect longer‐term outcomes, such as sepsis or organ dysfunction.

These factors all risk bias by indication, meaning that the patients receiving the exposure of interest, in this case RBC transfusion, are systematically different from those not receiving it (or receiving less of it) regarding the outcomes of interest. Bias by indication, due to inadequate correction for confounding variables, has been shown on multiple occasions to be a major limitation of observational blood transfusion research [26]. For example, many observational studies suggest transfusing RBCs stored for longer duration may worsen patient outcomes, but when randomised controlled trials were performed these provided no evidence of harm when compared with RBCs stored for a shorter duration [27]. More recently, methodologists have highlighted the need to consider the biological plausibility of effects in relation to time, particularly effects on outcomes that occur early which increases the probability they are explained by confounding.

Three systematic reviews addressed confounding adjustment in the primary studies [16, 17, 19], but not at the meta‐analysis level, and two reviews did not mention confounding adjustment at any level [15, 18]. These observations highlight the current uncertainty that the RBC exposure–outcome relationship for cancer surgery is causal. One approach to decreasing the risk of confounding in observational studies is propensity analyses. Of relevance, a recent review limiting studies to propensity‐adjusted observational studies in radical surgery for gastric and colorectal cancers found no association between RBC exposure and disease‐free survival [28]. We did not include this study in our review as some of the included studies did not specify only using RBCs as the blood product administered. However, the authors concluded that peri‐operative RBC transfusion was associated with worse overall survival which may be attributable to imbalances in the rates of major postoperative complications after propensity score adjustment.

Our study had strengths and limitations. Our protocol was registered prospectively, used rigorous data extraction methodology and quality assessment tools, and adhered to recommended umbrella review methodology. The strengths of umbrella reviews include structured evaluation of a topic based on published systematic reviews and meta‐analyses [29]. We summarised the broad and complex literature regarding RBC transfusion associations with cancer recurrence across different populations. However, limitations of this approach include the risk of missing studies not included in the included reviews, potential inclusion of individual studies across multiple included systematic reviews and limited quality assessment for the studies included in the systematic reviews. Our umbrella approach also meant we did not examine all the individual observational studies included in the systematic reviews. The risk of confounding is well‐recognised in observational studies in transfusion research [30]. We used group consensus, covering a range of medical specialities, to develop a framework to consider potential confounders to the exposure‐outcome relationships in the reviews. Although this approach could have missed important potential areas of confounding, it allowed a structured method to describe how this was addressed in the systematic reviews identified, alongside the AMSTAR 2 quality assessment tool. Exploring the quality of evidence and uncertainty about the association between transfusion and cancer recurrence, especially with contemporary blood products and transfusion practice, was the main aim of this umbrella review. Other limitations included a search strategy limited to full journal publications in English, which could have missed information in the grey literature, and variation in ascertaining and measuring cancer outcomes. Cancer recurrence and cancer‐free survival were measured in different ways and over different time periods in individual studies. Universal leukoreduction of all blood products was adopted in the UK in 1999 and across many countries in subsequent years. However, individual studies in the systematic reviews still used data from patients before implementation. As the studies include patient data from different countries, variation in leukoreduction practices and policy implementation mean there is inconsistency in blood products given to patients. Leukoreduction reduces the proinflammatory reaction associated with blood transfusion [31], which may theoretically lessen the impact of transfusion‐related immunomodulation. Even though all the included studies explicitly mentioned that they only included studies looking at RBC products exclusively, some of their included studies either do not specify which blood products were used or used a combination of blood products in their patients.

Our findings have implications for practice and future research. Our evidence summary shows significant uncertainty for there being a causal relationship between peri‐operative RBC transfusion and cancer recurrence, especially with contemporary surgical and oncology practice, and in an era of patient blood management systems. Although restrictive transfusion practice is recommended, a re‐evaluation of the risk‐to‐benefit relationship for some patients, for example those with severe anaemia and anaemia‐related symptoms (e.g. fatigue), or co‐existing morbidity (e.g. cardiovascular disease [32]) is warranted. Our proposed frameworks (Fig. 1 and Table 1) provide a list of potential confounders to adjust for in prospective studies, and many of these are collected routinely in healthcare records. Our review also highlights the need for high‐quality randomised controlled trials with relevant cancer‐related outcomes given the high risk of bias in observational research. A recent survey showed equipoise for a trial of transfusion thresholds in patients undergoing major abdominal cancer surgery [7].

In conclusion, peri‐operative RBC transfusion may be associated with adverse cancer‐related outcomes, but this is based on mostly critically low‐ and low‐quality evidence which adjusts for unmeasured confounding inadequately. Future observational studies should consider our framework for adjusting for confounders. Further research is required focusing on subgroups (e.g. severe anaemia and cardiovascular disease) and meaningful patient‐centred outcomes.

## Supporting information


**Appendix S1.** Search strategy.

**Table S1.** Summary of systematic review results regarding primary and secondary outcomes.
**Table S2.** Summary of risk of bias assessment, quality assessment and adjustment for confounding variables in included systematic reviews.
