# Supplementary material for: Association between peri‐operative red blood cell transfusion and cancer recurrence in patients undergoing major cancer surgery: an umbrella review*
Source: Anaesthesia. 2025 Jan 8;80(Suppl 2):65–74. doi: 10.1111/anae.16501 (PMC11744431; doi:10.1111/anae.16501)
Supplement: Supplementary file 1 — Appendix S1. Search strategy. [file ANAE-80-65-s001.docx]

**Appendix S1** Search strategy

The following databases were be searched for systematic reviews and meta-analyses”

- MEDLINE (ALL, Ovid, 1946 to present)
- PubMed (NLM, for epublications ahead of print only)
- Embase (Ovid, 1974 to present)
- CDSR, *The Cochrane Library* (2022, Issue 11)
- Epistemonikos (1990 to present)
- Transfusion Evidence Library (Evidentia, 1950 to present)

No date or language limits will be placed on the searches.

Searches retrieved 2,395 references and 1,425 references once duplicates had been removed.

**SEARCH STRATEGIES**

**MEDLINE**

1. exp Neoplasms/

2. (neoplas* or malignan* or tumo?r* or cancer* or carcinom* or adenocarcinom* or sarcoma* or hodgkin* or carcinosarcoma* or melanoma* or oncolog* or metasta* or allograft*).ti,kf.

3. 1 or 2

4. Blood Transfusion/

5. Blood Component Transfusion/

6. Erythrocyte Transfusion/

7. ((erythrocyte* or red blood cell* or red cell* or blood or RBC or PRBC*) adj3 (transfus* or unit*)).tw,kf.

8. ((transfus* or erythrocyte* or red cell* or red blood cell* or RBC or PRBC*) adj3 (trigger* or level* or threshold* or rule* or target* or restrict* or liberal* or requir* or reduc* or limit* or support* or management or sparing or strategy*)).tw,kf.

9. (blood adj3 (management or sparing or support* or strateg*)).tw,kf.

10. (hemotransfus* or haemotransfus* or hemotherap* or haemotherap*).tw,kf.

11. (red cell* or red blood cell* or RBC* or transfus*).ti.

12. or/4-11

13. Meta-Analysis/ or Network Meta-Analysis/

14. Systematic Review.pt.

15. "Systematic Reviews as Topic"/ or "Meta-Analysis as Topic"/

16. ((meta-analy* or metaanaly*) and (trials or studies)).ab.

17. (meta-analy* or metaanaly* or evidence-based).ti,kf.

18. ((systematic* or evidence-based) adj2 (review* or overview*)).tw,kf.

19. (network meta-analys* or network metaanalys* or evidence synthes* or cochrane or medline or pubmed or embase or cinahl or cinhal or lilacs or "web of science" or science citation index or scopus or search terms or literature search or electronic search* or comprehensive search* or systematic search* or published articles or search strateg* or reference list* or bibliograph* or handsearch* or hand search* or manual* search*).ab.

20. Cochrane Database of systematic reviews.jn.

21. ((additional adj (papers or articles or sources)) or (relevant adj (journals or articles))).ab.

22. ((electronic* or online) adj (sources or resources or databases)).ab.

23. or/13-22

24. Review.pt.

25. Randomized Controlled Trials as Topic/

26. selection criteria.ab. or critical appraisal.ti.

27. (data adj (abstraction or extraction or analys*)).ab.

28. exp Randomized Controlled Trial/

29. or/25-28

30. 24 and 29

31. 23 or 30

32. 3 and 12 and 31

**PubMed**

#1 (neoplas*[TI] OR malignan*[TI] OR tumor*[TI] OR tumour*[TI] OR cancer*[TI] OR carcinom*[TI] OR adenocarcinom*[TI] OR sarcoma*[TI] OR hodgkin*[TI] OR carcinosarcoma*[TI] OR melanoma*[TI] OR oncolog*[TI] OR metasta*[TI] OR allograft*[TI])

#2 (transfus*[TIAB] OR ((erythrocyte*[TI] OR "red cell*"[TI] OR blood[TI] OR RBC[TI] OR RBCs[TI]) AND (unit*[TI] OR trigger*[TI] OR level*[TI] OR target*[TI] OR threshold*[TI] OR rule*[TI] OR restrict*[TI] OR liberal*[TI] OR requir*[TI] OR reduc*[TI] OR limit*[TI] OR support*[TI] OR management[TI] OR sparing[TI] OR strateg*[TI])) OR hemotransfus*[TIAB] OR haemotransfus*[TIAB] OR hemotherap*[TIAB] OR haemotherap*[TIAB])

#3 ("systematic review"[TIAB] OR "systematic overview" [TIAB] OR "meta-analysis" OR metaanalysis[TIAB] OR "evidence synthesis"[TIAB] OR "literature search"[TIAB] OR medline[TIAB] OR pubmed[TIAB] OR cochrane[TIAB] OR embase[TIAB] OR scopus[TIAB] OR "web of science"[TIAB]) NOT medline[sb]

#4 #1 AND #2 AND #3

**Embase**

1. exp *Malignant Neoplasm/

2. (neoplas* or malignan* or tumo?r* or cancer* or carcinom* or adenocarcinom* or sarcoma* or hodgkin* or carcinosarcoma* or melanoma* or oncolog* or metasta* or allograft*).ti,kf.

3. 1 or 2

4. *Blood Transfusion/

5. Blood Component Transfusion/

6. Erythrocyte Transfusion/

7. ((erythrocyte* or red blood cell* or red cell* or blood or RBC*) adj3 (transfus* or unit*)).tw,kf.

8. ((transfus* or erythrocyte* or red cell* or red blood cell* or RBC*) adj3 (trigger* or level* or threshold* or rule* or target* or restrict* or liberal* or requir* or reduc* or limit* or support* or management or sparing or strateg*)).tw,kf.

9. (blood adj3 (management or sparing or support* or strateg*)).tw,kf.

10. (hemotransfus* or haemotransfus* or hemotherap* or haemotherap*).tw,kf.

11. (red cell* or red blood cell* or RBC* or transfus*).ti.

12. or/4-11

13. exp Meta Analysis/ or "Meta Analysis (Topic)"/

14. (meta-analy* or metaanaly* or evidence-based).ti.

15. ((meta-analy* or metaanaly*) and (trials or studies)).ab.

16. Systematic Review/ or "Systematic Review (Topic)"/

17. ((systematic* or evidence-based) adj2 (review* or overview*)).tw.

18. (network meta-analys* or evidence synthes* or cochrane or medline or pubmed or embase or cinahl or cinhal or lilacs or "web of science" or science citation index or scopus or search terms or literature search or electronic search* or comprehensive search* or systematic search* or published articles or search strateg* or reference list* or bibliograph* or handsearch* or hand search* or manual* search*).ab.

19. ((electronic* or online) adj (sources or resources or databases)).ab.

20. ((additional adj (papers or articles or sources)) or (relevant adj (journals or articles))).ab.

21. Review.pt. and (data extraction or selection criteria).ab.

22. or/13-21

23. Editorial.pt.

24. 22 not 23

25. 3 and 12 and 24

**CENTRAL**

#1 MeSH descriptor: [Neoplasms] explode all trees

#2 (neoplas* or malignan* or tumo?r* or cancer* or carcinom* or adenocarcinom* or sarcoma* or hodgkin* or carcinosarcoma* or melanoma* or oncolog* or metasta* or allograft*):ti

#3 #1 or #2

#4 MeSH descriptor: [Blood Transfusion] this term only

#5 MeSH descriptor: [Blood Component Transfusion] this term only

#6 MeSH descriptor: [Erythrocyte Transfusion] this term only

#7 ((erythrocyte* or red blood cell* or red cell* or blood or RBC*) near/3 (transfus* or unit*)):ti,ab

#8 ((transfus* or erythrocyte* or red cell* or red blood cell* or RBC*) near/3 (trigger* or level* or threshold* or rule* or target* or restrict* or liberal* or requir* or reduc* or limit* or support* or management or sparing or strateg*)):ti,ab

#9 (blood near/3 (management or sparing or support* or strateg*)):ti,ab

#10 (hemotransfus* or haemotransfus* or hemotherap* or haemotherap*)

#11 (red cell* or red blood cell* or RBC* or transfus*):ti

#12 #4 or #5 or #6 or #7 or #8 or #9 or #10 or #11

#13 #3 and #12 in Cochrane Reviews

**EPISTEMONIKOS**

(title:(neoplas* OR malignan* OR tumor* OR tumour* OR cancer* OR carcinom* OR adenocarcinom* OR sarcoma* OR hodgkin* OR carcinosarcoma* OR melanoma* OR oncolog* OR metasta* OR allograft*) AND ((title:(transfus*) OR abstract:(transfus*) OR title:((erythrocyte* OR "red cell" OR "red cells" OR blood OR RBC OR RBCs) AND (unit* OR trigger* OR level* OR target* OR threshold* OR rule* OR restrict* OR liberal* OR requir* OR reduc* OR limit* OR support* OR management OR sparing OR strateg*)) OR hemotransfus* OR haemotransfus* OR hemotherap* OR haemotherap*)))

**TRANSFUSION EVIDENCE LIBRARY**

(Subject Area: Red Cells OR (title:(neoplasm OR neoplasms OR malignant OR malignancy OR malignancies OR tumor OR tumour OR cancer OR carcinoma OR adenocarcinoma OR sarcoma OR hodgkin OR carcinosarcoma OR melanoma OR oncology OR oncologic OR metastatic OR metastasis OR metastases OR allograft OR allografts) AND title:(transfusion OR transfusions OR transfused OR erythrocyte OR erythrocytes OR red cell OR red cells OR red blood cell OR red blood cells OR RBC OR RBCs)))

AND Study Type: Systematic Reviews
