# Supplementary material for: Association between peri‐operative red blood cell transfusion and cancer recurrence in patients undergoing major cancer surgery: an umbrella review*
Source: Anaesthesia. 2025 Jan 8;80(Suppl 2):65–74. doi: 10.1111/anae.16501 (PMC11744431; doi:10.1111/anae.16501)
Supplement: Supplementary file 2 — Table S1. Summary of systematic review results regarding primary and secondary outcomes. Table S2. Summary of risk of bias assessment, quality assessment and adjustment for confounding variables in included systematic reviews. [file ANAE-80-65-s002.docx]

**Table S1** – Summary of systematic review results regarding primary and secondary outcomes

| **First author** | **Cancer recurrence** | | | **Recurrence free survival** | | | **All-cause mortality/overall survival** | | | **Cancer related mortality/Disease-free survival(DFS)** | | | **Recurrence-metastasis-death** | | | **Other outcomes** | | |
| --- | --- | --- | --- | --- | --- | --- | --- | --- | --- | --- | --- | --- | --- | --- | --- | --- | --- | --- |
|  | Intervention | Control | OR (95% CI) | Intervention | Control | OR (95% CI) | Intervention | Control | OR (95% CI) | Intervention | Control | OR (95% CI) | Intervention | Control | OR (95% CI) | Intervention | Control | OR (95% CI) |
| Vamvakas [18] | Colorectal – Risk ratio 1.49 (95% CI 1.23-1.79)  Breast – Relative Risk1.06 (95% CI 0.90-1.24)  Head and neck – Relative Risk 3.62 (95% CI 2.15-6.08)    Lung – Relative Risk 1.30 (95% CI 1.02-1.66)  Prostate – Relative Risk 1.51 (95% CI 1.13-2.01)  Gastric – Relative Risk 2.44 (95% CI 1.60-3.71) | | |  |  |  |  | | |  | | |  |  |  |  |  |  |
| Amato [16] | 363/1016 (35.7%) | 288/1094 (26.3%) | 1.65 (1.35-2.01) |  |  |  |  | | |  | | |  |  |  |  |  |  |
| Acheson [15] |  |  |  |  |  |  | 3010/6751 (44.6%) | | | 1644/4744 (34.7% | | | 1.72 (1.55-1.91) | 1252/4045 (31.0%) | 638/2614 (24.4%) | 1.71 (1.43-2.05) | 1867/4336 (43.1%) | 961/2889 (33.3%) |
| Bennett [19] |  |  |  |  |  |  | One of five studies (20%) demonstrate increased mortality following transfusion | | | Ten of eighteen studies (55.6%) demonstrate decreased cancer related survival (OS and DFS) following transfusion | | |  |  |  | Five of six studies demonstrate increased post-operative complications following transfusion |  |  |
| Petrelli [17] |  |  |  |  |  |  | OSS: Hazard ratio 1.50 (95% CI 1.42-1.57) | | | DFS: Hazard ratio 1.36 (95% CI 1.26-1.46) | | |  |  |  |  |  |  |

**Table S2** – Summary of risk of bias assessment, quality assessment and adjustment for confounding variables in included systematic reviews

| **First author** | **Was a risk of bias tool used?** | **Risk of bias score** | **Was a quality assessment used by the authors?** | **Quality assessment score average (/maximum score)** | **Identification of confounding variables** | **Adjustment made for confounding variables** |
| --- | --- | --- | --- | --- | --- | --- |
| Vamvakas [18] | No | N/A | No | - | Study design, sample size, definition of transfusion exposure and outcome measures, publication year, type of surgery, and patient characteristics | No |
| Amato [16], A | Author’s judgement | 3 = low risk  7 = unclear risk | Evans and Pollock system for evaluating random control clinical trials | 66 / 100 | Patient demographics (e.g., age, sex) and comorbidities, pre-operative anaemia, duration of operation, volume of blood loss, and amount, type, and timing of perioperative blood transfusion | Multivariate techniques from primary studies described qualitatively, but no further adjustment during meta-analysis |
| Acheson [15] | No | N/A | NOS | 7.36 / 9 | Age, tumour localisation, Duke’s staging, pre-operative haemoglobin levels, presence of pre-operative anaemia, and volume of blood loss during surgery. | No |
| Bennett [19 | ACROBAT-NRSI | 0 = Low risk  18 = Moderate risk  1 = Serious risk  3 = Critical risk’ | No | - | Surgical technique, patient age, patient sex, comorbidities, volume of blood loss, tumour characteristics (size, grade, etc.), vascular invasion, length of operation, year of operation, extent of resection, and other tumour specific variables (e.g., LFTs, Child-Pugh classification, anti-HCV Ab). | Adjustments made at level of primary studies, but not during systematic review |
| Petrelli [17] | Author’s judgement | Low risk = 45.5%  Medium risk = 37.4%  High risk = 17.9 | NOS | 6.40 / 9 | Age, patient sex, cancer stage, haemoglobin levels, American Society of Anesthesiology (ASA) score, and performance status | Adjustments made at level of some of the primary studies (not all), but not during meta-analysis |

NOS – Newcastle Ottawa score; ACROBAT-NRSI, A Cochrane Risk Of Bias Assessment Tool for Non-Randomized Studies of Interventions.
